# 1 / 1 Exploring the Clinical and Psychosocial Predictors of Suicidal Ideation in Patients with Systemic Lupus Erythematosus

**DOI:** 10.1192/j.eurpsy.2025.1250

**Published:** 2025-08-26

**Authors:** M. Recio-Barbero, J. Cabezas-Garduño, J. Varona, G. Ruiz-Irastorza, S. Sanz-Gómez, M. Shahine, A. Lengvenyte, P. Courtet, R. Segarra

**Affiliations:** 1 Department of Psychiatry. Cruces University Hospital; 2 Autoimmune Diseases Research Unit, Internal Medicine Department, Biobizkaia Health Research Institute, Barakaldo; 3 Departamento de Psiquiatría, Universidad de Sevilla, Sevilla, Spain; 4 Univ. Montpellier, CNRS, INSERM, IGF; 5 Department of Emergency Psychiatry and Acute Care, Lapeyronie Hospital, CHU Montpellier, IGF, Univ. Montpellier, CNRS, INSERM, Montpellier, France; 6 CIBERSAM ISCIII, Centro de Investigación Biomédica en Red de Salud Mental, Leioa, Spain

## Abstract

**Introduction:**

Systemic Lupus Erythematosus (SLE) is an autoimmune disorder characterized by systemic inflammation and high prevalence of neuropsychiatric symptoms. Among these, suicidal ideation represents a significant concern, with reported higher rates than in the general population. The etiology of suicidal ideation in SLE is multifaceted, encompassing both disease-specific factors and psychosocial variables. Recent research has highlighted the intricate interplay between SLE and psychiatric symptoms, suggesting a bidirectional relationship mediated by inflammatory processes, neurological involvement, and psychosocial stressors. However, the specific contributions of these factors remain unclear.

**Objectives:**

This study aims to elucidate the primary predictors of suicidal ideation in a cohort of SLE patients.

**Methods:**

A cross-sectional study was conducted with a cohort of SLE patients from the Autoimmune Diseases Unit at Cruces University Hospital (Biscay, Spain). Each patient underwent a thorough clinical evaluation by a psychiatrist, and relevant clinical and sociodemographic data were collected. Suicidal ideation was assessed using the Plutchik scale, while predictor variables included autoimmune factors such as cumulative corticosteroid dose, disease activity, and accumulated organ damage. Psychiatric symptoms were assessed using various psychometric scales. A multiple linear regression model was employed to examine the associations between these variables and suicidal ideation.

**Results:**

A total of 92 SLE patients in active follow-up were included, with a female predominance (91%). Most patients were in an inactive disease state, receiving low-dose prednisone and antimalarial therapy according to our clinical protocol; the majority were in remission, as reflected by a mean SLEDAI-2K score of 1.59(±2.44). Despite a median disease duration of over 10 years, cumulative organ damage was low (mean SDI score 0.33 ± 0.84). In a multiple linear regression analysis, the model showed a strong fit (R=0.732, R²=0.535, p<.001), explaining approximately 53.5% of the variability in suicidal ideation. Suicidal ideation was significantly associated with cumulative corticosteroid dose (β=0.172, p=0.011), total anxiety score (β=0.248, p<.001), and frequency of traumatic experiences (β=0.118, p<.001). Collinearity statistics (VIF 1.03–1.07) indicated no significant multicollinearity issues.

**Conclusions:**

Suicidal ideation in our SLE cohort was associated with corticosteroid use, anxiety, and traumatic experiences, highlighting the importance of addressing these factors in suicide risk management. The relationship between corticosteroid dose and suicidal ideation may be linked to the neuropsychiatric effects of these drugs, while anxiety and traumatic experiences contribute to the emotional burden of the patient. Notably, disease activity was not linked to suicidal ideation in this cohort.

**Disclosure of Interest:**

None Declared

